# Treatment strategy changes for inflammatory bowel diseases in biologic era: results from a multicenter cohort in Japan, Far East 1000

**DOI:** 10.1038/s41598-023-40624-5

**Published:** 2023-08-21

**Authors:** Takashi Taida, Yuki Ohta, Jun Kato, Sadahisa Ogasawara, Yuhei Ohyama, Yukiyo Mamiya, Hayato Nakazawa, Ryosuke Horio, Chihiro Goto, Satsuki Takahashi, Akane Kurosugi, Michiko Sonoda, Wataru Shiratori, Tatsuya Kaneko, Yuya Yokoyama, Naoki Akizue, Yotaro Iino, Junichiro Kumagai, Hideaki Ishigami, Hirotaka Koseki, Kenichiro Okimoto, Keiko Saito, Masaya Saito, Tomoaki Matsumura, Tomoo Nakagawa, Shinichiro Okabe, Hirofumi Saito, Kazuki Kato, Hirotsugu Uehara, Hideaki Mizumoto, Yoshihiro Koma, Ryosaku Azemoto, Kenji Ito, Hidehiro Kamezaki, Yoshifumi Mandai, Yoshio Masuya, Yoshihiro Fukuda, Yoshio Kitsukawa, Haruhisa Shimura, Toshio Tsuyuguchi, Naoya Kato

**Affiliations:** 1 Department of Gastroenterology, Graduate School of Medicine, Chiba University, 1-8-1 Inohana, Chuo-ku, Chiba, 260-8670 Japan; 2 Endoscopy Center, Chiba University Hospital, Chiba, Japan; 3 Department of Gastroenterology, Chibaken Saiseikai Narashino Hospital, Narashino, Japan; 4 Department of Gastroenterology, Kimitsu Chuo Hospital, Kisarazu, Japan; 5 Department of Gastroenterology, Chiba Rosai Hospital, Chiba, Japan; 6 Department of Internal Medicine, Chiba Aoba Municipal Hospital, Chiba, Japan; 7 Department of Gastroenterology, Seikei-Kai Chiba Medical Center, Chiba, Japan; 8 Department of Gastroenterology, Matsudo City General Hospital, Matsudo, Japan; 9 Department of Gastroenterology, Chiba Kaihin Municipal Hospital, Chiba, Japan; 10 Department of Gastroenterology, Funabashi Central Hospital, Funabashi, Japan; 11 Department of Gastroenterology, Chiba Central Medical Center, Chiba, Japan; 12 Department of Gastroenterology, Funabashi Municipal Medical Center, Funabashi, Japan; 13 Department of Gastroenterology, Chiba Medical Center, Chiba, Japan; 14 Department of Gastroenterology, Eastern Chiba Medical Center, Togane, Japan; 15 Department of Gastroenterology, Japanese Red Cross Narita Hospital, Narita, Japan; 16 Department of Gastroenterology, Asahi General Hospital, Asahi, Japan

**Keywords:** Gastrointestinal diseases, Epidemiology

## Abstract

Many molecular targeted agents, including biologics, have emerged for inflammatory bowel diseases (IBD), but their high prices have prevented their widespread use. This study aimed to reveal the changes in patient characteristics and the therapeutic strategies of IBD before and after the implementation of biologics in Japan, where the unique health insurance system allows patients with IBD and physicians to select drugs with minimum patient expenses. The analysis was performed using a prospective cohort, including IBD expert and nonexpert hospitals in Japan. In this study, patients were classified into two groups according to the year of diagnosis based on infliximab implementation as the prebiologic and biologic era groups. The characteristics of therapeutic strategies in both groups were evaluated using association analysis. This study analyzed 542 ulcerative colitis (UC) and 186 Crohn’s disease (CD). The biologic era included 53.3% of patients with UC and 76.2% with CD, respectively. The age of UC (33.9 years vs. 38.8 years, *P* < 0.001) or CD diagnosis (24.3 years vs. 31.9 years, *P* < 0.001) was significantly higher in the biologic era group. The association analysis of patients with multiple drug usage histories revealed that patients in the prebiologic era group selected anti-tumor necrosis factor (TNF)-α agents, whereas those in the biologic era group preferred biologic agents with different mechanisms other than anti-TNF-α. In conclusion, this study demonstrated that both patient characteristics and treatment preferences in IBD have changed before and after biologic implementation.

## Introduction

Inflammatory bowel disease (IBD) is an intractable gastrointestinal disorder without radical treatment and is characterized by chronic repeated relapses and remissions^1^. The number of patients with IBD has been recently increasing in Asia as previously observed in Europe and the United States^2^. In Japan, both ulcerative colitis (UC) and Crohn’s disease (CD) are increasing, and the number of patients with UC is the second largest in the world in the United States. Hence, IBD in Japan has recently evolved from rare diseases that were treated only in specialized hospitals to common diseases that are seen in community hospitals. Accordingly, quite a few nonspecialist physicians are now engaged in the management of many patients with IBD in Japan.

IBD treatment has drastically changed over the past decades since the approval of several antitumor necrosis factor (TNF)-α agents following the report on the induction of remission for patients with CD in 1997^3,4^. Recent progress in immunological studies has led to the approval of drugs with different mechanisms of action, such as ustekinumab (UST)^5^, vedolizumab (VED)^6^, and tofacitinib (TOF)^7^, which have further expanded the scope of IBD treatment strategy. However, the cost of these new drugs is a major medical issue and their use greatly varies depending on the individual patient’s medical environment^8^. Additionally, data on the appropriate treatment selection for patients with refractory IBD remained lacking.

Clinical practice guidelines for IBD treatment in Japan have demonstrated reasonable and simplified treatment strategies^9^, as well as guidelines in Europe^10,11^ and the US^12,13^. Moreover, the costs of agents have little impact on treatment selection in Japan as a large portion of medical costs are covered under the universal health insurance system. In particular, the majority of medical costs even biologics are subsidized^14,15^ due to the special subsidy system for intractable diseases for designated intractable diseases, including IBD. Therefore, Japan’s unique medical systems generally allow the treatment selection of biologic agents according to disease status with minimum consideration of economic issues.

The application of data mining of patients with IBD has become a topic of great interest^16^. Drug selection and the combination of different types of agents are determined by physicians in medical therapy, based on the distinct information on the disease condition of each patient and interactions between drugs. Therefore, the trend of treatment patterns for patients with IBD could be analyzed using the data mining method. However, there are few reports on mining treatment patterns in IBD medication.

This study aimed to reveal the characteristics and therapeutic strategies before and after the implementation of biologics in Japanese real-world practice by conducting data mining. Our analysis may reveal patients’ and physicians’ real medication preferences because of the situation of almost free access to expensive biologics due to the unique subsidy system in Japan. The data used in this analysis were obtained from the prospective cohort titled “Features in Japanese Patients with Inflammatory Bowel Diseases: A multicenter prospective long observation study (Far East 1000).” This cohort, notably, included both IBD-specialized hospitals and community hospitals without IBD specialists to clarify the whole picture of the trend of IBD medications.

## Materials and methods

### Study design and participants

This study analyzed the data of patients with IBD enrolled in a prospectively collected cohort. The Far East 1000 is a multicenter cohort for long-term investigation of disease activity, treatment strategy, and complications of IBD. The details of this cohort with the results of the prevalence of extraintestinal manifestations were previously reported^17^. The participating hospitals included 15 major medical institutions (two hospitals with < 300 beds, eight hospitals with 300–500 beds, two hospitals with 500–700 beds, and three hospitals with > 700 beds) in Chiba, covering approximately 6.15 million inhabitants (Supplementary Figure 1). Of 15 participating hospitals, 5 are expert hospitals specialized in IBD care (with a total of 2890 beds) and the remaining 10 were nonexpert community hospitals (with a total of 4631 beds), enabling actual IBD situation clarification. Expert hospitals is defined as facilities with physicians who have practiced IBD for ≥ 3 years and have expertise in the use of biologics and immunosuppressive agents. All of the institutions are staffed by gastroenterologists and provide guideline-compliant care.

Chiba encompasses the Boso Peninsula, which is surrounded by the sea, and its geography allows the long-term follow-up of patients due to minimum patient mobility. The Far East 1000 cohort was launched in January 2019 based on this geographic feature.

The cohort collects the following medical information: gender; age at onset and age at enrollment diagnosis; IBD disease distribution; smoking habit; disease activity, such as Lichtiger index in UC and clinical disease activity index in CD; laboratory tests; endoscopic scores, such as Mayo endoscopic subscore in UC and simple-endoscopic score for Crohn’s disease (SES-CD) in CD; and the current and past medications, including 5-aminosalicylic acid (5-ASA), corticosteroids, immunomodulators, biologics/JAK inhibitors, apheresis, herbal drugs, elemental diet, and probiotics. Data were obtained on each patient’s entire clinical course from the time of diagnosis to enrollment regarding the aforementioned parameters. Patient information was obtained from the medical charts, as well as a specific questionnaire, after obtaining informed consent.

This study analyzed patients with UC or CD who were enrolled in this cohort from January 2019 to September 2019. The data of this study was locked on September 30, 2019. This study was conducted in accordance with the "Ethical Guidelines for Medical Research Involving Human Subjects" established by the Ministry of Education, Culture, Sports, Science and Technology and the Ministry of Health, Labour and Welfare of Japan, and approved by the Research Ethics Committee of Chiba University Hospital (Approval no. 3107).

### Assessment of treatment strategies for IBD

IBD treatment was provided based on the Japanese evidence-based clinical practice guidelines^9^. The medication was selected according to disease severity and corticosteroid response after UC or CD diagnosis. Access to all therapies, including all types of biologics for moderate to severe cases, is generally guaranteed due to medical subsidy.

This study divided patients into two groups according to the time of IBD diagnosis: the biologic and prebiologic era groups. Biologics became available with the approval of infliximab (IFX) in 2002 for CD and in 2010 for UC in Japan. Various biologics were approved since IFX approval, and treatment strategies have drastically changed in the fields of both UC and CD. Therefore, patients diagnosed with CD on or after 2002 and UC on or after 2010 were classified into the biologic era group. Patients diagnosed before the biologic era group were classified into the prebiologic era group.

### Association analysis

This study used the association analysis to assess the evolution in treatment strategy with biological application associated with drug selection preference, about previous reports analyzing other diseases^18,19^. This analysis is a data mining technique to investigate frequent co-occurring associations among variables in large databases, which means the analysis reveals which drugs are more likely to be selected with a history of multiple drug use, not the frequency of each drug.

Association rules were created by specifying treatments as antecedent and consequent ones so that the reverse associations were not detected. The association was measured using three parameters: “support” (how frequently the treatment combination appears in the dataset), “confidence” (the conditional probability that a subject with the antecedent treatment will also have the consequent treatment), and “lift” (the ratio of the observed support to that expected if the two events were independent). The minimum parameter thresholds for “support” and “confidence” were set at 0.001 and 0.5 to extract a sufficient number of association rules, respectively.“Lift” is the key parameter in the association analysis and indicates the correlation between the likelihood of a specific condition and that of other events. This study used association analysis to evaluate the influence of certain treatment histories on the choice of a particular medication. Specifically, a “lift” of > 1.0 indicates that the antecedent and consequent treatments appear more often than expected, i.e., the initial treatment exerts a positive effect on the consequent treatment occurrence. The association analysis was performed on the full cross-sectional dataset, and separately for the prebiological and biological subgroups. The preference patterns of medications for UC and CD were compared between the patient groups.

### Statistical analysis

Demographic and clinical characteristics were compared using the Chi-square test or Fisher’s exact test. Continuous data were analyzed using Spearman’s rank correlation or the Mann–Whitney U test. These statistical analyses were performed using Statistical Package for the Social Sciences version 25.0 (SPSS Inc., Chicago, IL, USA) and Prism version 9.3.1 (GraphPad Software, LCC.). A *P* value of < 0.05 was considered statistically significant. The association analysis for mining data and the discovery of the interesting relationships in treatment was performed with arules package (v1.6–8) in R (v4.0.3).

### Disclosure

 Sadahisa Ogasawara received honoraria from Eisai Co., Ltd., Takeda Pharmaceutical Co., Ltd., Abbvie G.K.; consulting or advisory fees from Eisai Co., Ltd., and research grants from Eisai Co., Ltd.. Naoya Kato received honoraria from AbbVie G.K., Ohtsuka Pharmaceutical Co., Ltd., Takeda Pharmaceutical Co., Ltd., Zeria Pharmaceutical Co., Ltd., Olympus Corporation, Eisai Co., Ltd., Tsumura & Co., Mochida Pharmaceutical Co., Ltd., Miyarisan Pharmaceutical Co., Ltd., Yakult Honsha Co., Ltd., Olympus Marketing, Inc., and Janssen Pharmaceutical K.K., and research funding from Ohtsuka Pharmaceutical Co., Ltd., Mitsubishi Tanabe Pharma Corporation, Eisai Co., Ltd., Tsumura & Co., Nippon Kayaku Co., Ltd., and JIMRO Co., Ltd. The other authors have no conflicts of interest to declare.

## Results

### Study population

**Figure 1:**
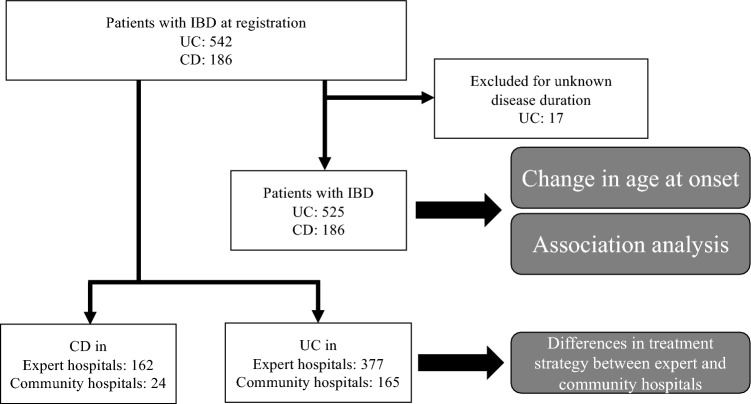
The workflow of the study. The analysis of change in age at onset and association analysis in treatment selection was performed.

The workflow of this study was shown in Fig. 1. The Far East 1000 cohort registered 728 patients, including 542 with UC and 186 with CD, from January 2019 to September 2019 (Supplementary Figure 2 shows the year of diagnosis of patients with UC and CD). The proportions of patients diagnosed in the prebiologic and biologic era were 46.7% and 53.3% for UC, and 23.8% and 76.2% for CD, respectively.

Baseline characteristics of the cohort were shown in Table 1. The mean age at diagnosis was 36.5 years for UC and 30.1 years for CD, and the patients had been treated for > 10 years on average before cohort enrollment. Approximately half of the UC cases had pancolitis, and approximately half of CD cases had ileocolonic diseases. At the time of enrollment, 18 (3.3%) patients with UC and 79 (42.5%) patients with CD had a history of surgery. Intestinal complications were present in 107 (57.5%) patients with CD, especially intestinal stricture in 95 (51.1%) patients. Anal lesions were present in 50 patients (26.9%). Surgery for intestinal complications in CD was performed in 63 cases (33.9%).

**Table 1:** Demographic and clinical characteristics of patients with ulcerative colitis and Crohn’s disease.

	Any IBD	UC	CD
Number of patients	728	542 (74.5%)	186 (25.5%)
Gender, male (n [%])	435 (59.75%)	300 (55.35%)	135 (72.58%)
Age at the time of onset, years, Mean (SD)	33.8 (15.4)	36.0 (15.3)	27.5 (14.1)
Age at the time of diagnosis, years, Mean (SD)	34.8 (15.4)	36.5 (15.2)	30.1 (15.2)
Disease duration, years, Mean (SD)	11.7 (9.72)	11.7 (9.96)	11.6 (9.02)
History of Surgery (n [%])	97 (13.3%)	18 (3.3%)	79 (42.5%)
*Type of IBD at the time of participation in this study*			
Pancolitis (n [%])		244 (45.02%)	
Left-sided colitis (n [%])		147 (27.12%)	
Proctitis (n [%])		89 (16.42%)	
Right-sided or segmental colitis (n [%])		7 (1.29%)	
Unknown (n [%])		52 (9.59%)	
Ileal (*n* [%])			41 (22.04%)
Colic (*n* [%])			28 (15.05%)
Ileocolic (*n* [%])			102 (54.84%)
Unknown (*n* [%])			15 (8.06%)
*Smoking*			
None (*n* [%])	372 (51.10%)	275 (50.74%)	97 (52.15%)
Previous smoker (*n* [%])	89 (12.22%)	58 (10.70%)	31 (16.67%)
Current smoker (*n* [%])	260 (35.71%)	203 (37.45%)	57 (30.65%)
Unknown (*n* [%])	7 (0.96%)	6 (1.11%)	1 (0.54%)

**Figure 2:**
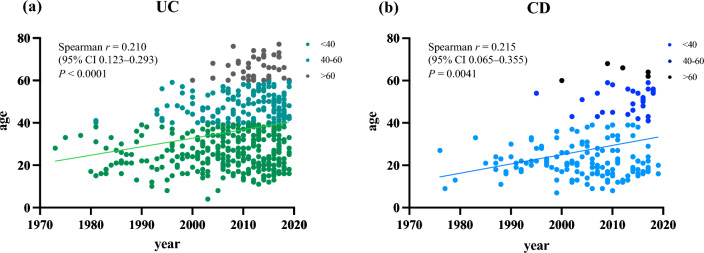
Changes in age at onset. The age at onset of both ulcerative colitis (UC) (**a**) and Crohn’s disease (CD) (**b**) has increased over time.

Figure 2 shows the age at disease onset according to the calendar year of onset. Most patients with UC and CD with onset from the 1970s to the 1990s developed the disease at a young age, but patients with older onset have increased since the 2000s. Significantly positive correlations were found between the onset year and age at diagnosis of UC (Spearman’s correlation, *r* = 0.210, *P* < 0.0001) and CD (Spearman’s correlation, *r* = 0.215, *P* = 0.0041), respectively.

### Differences in treatment strategy in patients with UC and CD between expert hospitals and community hospitals

**Figure 3:**
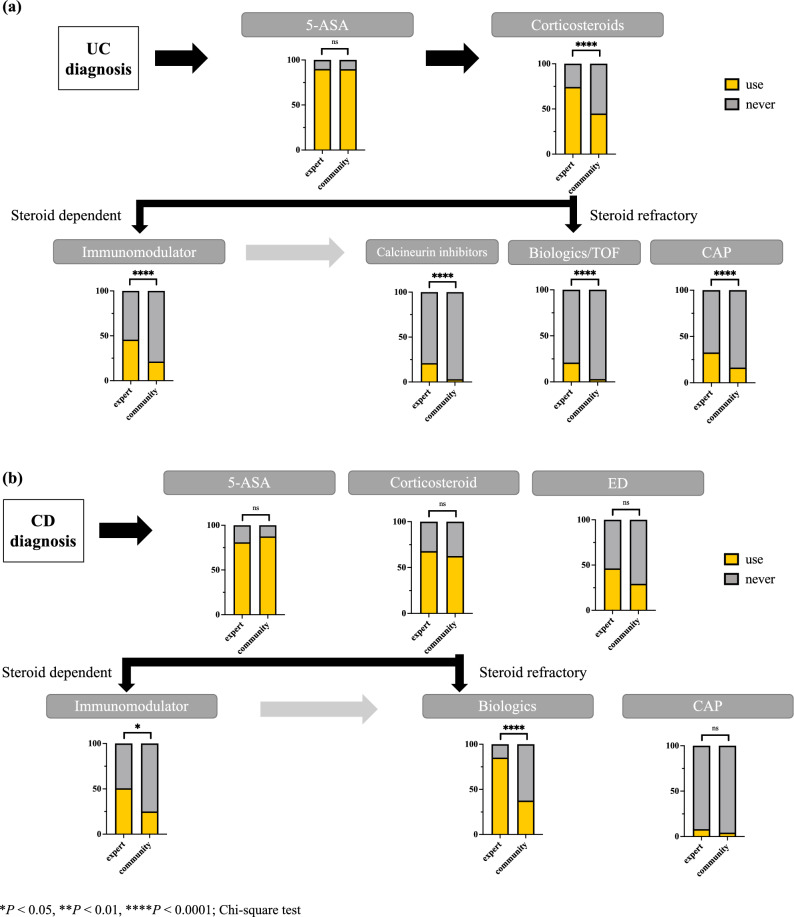
Proportion of patients with inflammatory bowel diseases (IBD) who have been prescribed each mediation in expert hospitals versus nonexpert community hospitals. (**a**) The proportion of patients with UC who have been prescribed each mediation in expert hospitals versus nonexpert community hospitals. The treatment strategies shown in the figure are based on the Japanese treatment guidelines for UC^9^. **(b)** The proportion of patients with CD who have been prescribed each mediation in expert hospitals versus nonexpert community hospitals. The treatment strategies shown in the figure are based on the Japanese treatment guidelines for CD^9^.

In this cohort, 165 (30.4%) patients with UC and 24 (12.9%) with CD were treated at nonexpert community hospitals, while the remaining 377 (69.6%) with UC and 162 (87.1%) with CD were treated at expert hospitals. Figure 3 shows the percentage of patients in each hospital group (UC: Fig. 3a, CD: Fig. 3b) who have used each medication since disease onset. Therapeutics, except 5-ASA, particularly immunomodulators and biologics, were predominantly prescribed in expert hospitals both in patients with UC and CD.

### Change of features of patients with IBD from prebiologic era to biologic era

Comparisons between patients diagnosed in the prebiologic and those in the biologic era are shown in Table 2 (UC) and 3 (CD). The age at UC diagnosis was significantly higher in the biologic era group (33.9 years in the prebiologic era vs. 38.8 years in the biologic era, *P* < 0.001). Clinical activity at diagnosis did not differ regardless of the diagnostic time point; however, the use of biologics/JAK inhibitor was more frequent in the biologic era group despite the shorter treatment duration (21.6% in prebiologic era vs. 32.1% in biologic era, *P* = 0.007). Among biologic and small molecule drugs, patients of the biologic era group were relatively more likely administered novel agents, such as golimumab (GLM) (*P* = 0.006), vedolizumab (VED) (*P* = 0.138), and tofacitinib (TOF) (*P* = 0.069). Patients of the prebiologic era group were significantly more likely to receive apheresis therapy (*P* = 0.014).

**Table 2:** Comparisons between patients with UC diagnosed in the prebiologic and biologic era.

	Prebiologic era	Biologic era	*P* value
Number of patients	44 (23.7%)	142 (76.3%)	
Gender, male (n [%])	33 (75.0%)	102 (71.8%)	0.681
Age at the time of diagnosis, years, Mean (SD)	24.3 (10.6)	31.9 (16.0)	< 0.001
Disease duration, years, Mean (SD)	24.8 (6.0)	7.4 (4.9)	< 0.001
CDAI at diagnosis, Mean (SD)	72.5 (62.7)	44.2 (45.5)	0.003
Treatment			
Biological treatment	36 (81.8%)	110 (77.5%)	0.539
IFX (n [%])	30 (68.2%)	74 (52.1%)	0.061
IFX-BS (n [%])	4 (9.1%)	32 (22.5%)	0.049
ADA (n [%])	12 (27.3%)	45 (31.7%)	0.579
UST (n [%])	2 (4.5%)	14 (9.9%)	0.272
Other treatment			
5-ASA (n [%])	40 (90.9%)	111 (78.2%)	0.059
Corticosteroids	23 (52.2%)	59 (41.5%)	0.191
Immunomodulator (n [%])	17 (38.6%)	70 (49.3%)	0.245
Apheresis (n [%])	2 (4.5%)	12 (8.5%)	0.385
Probiotics (n [%])	28 (64.6%)	88 (62.0%)	0.801
Elemental diet (n [%])	24 (54.5%)	63 (44.4%)	0.111
Dose of elemental diet, kcal (SD)	806.3 (389.9)	660.3 (285.4)	
Herbal drug (n [%])	2 (4.5%)	10 (7.0%)	0.579

The age at CD diagnosis was also significantly higher in the biologic era group (24.3 years in the prebiologic era group vs. 31.9 years in the biologic era group, *P* < 0.001). Biologics were used in approximately 80% of patients, without significantly different frequency between patients of the prebiologics era group and those of the biologics era group (81.8% vs. 77.5%, *P* = 0.539) (Table 3).

**Table 3:** Comparisons between patients with CD diagnosed in the prebiologic and biologic era.

	Prebiologic era	Biologic era	*P* value
Number of patients	245 (45.2%)	280 (51.7%)	
Gender, male (n [%])	133 (54.3%)	158 (56.4%)	0.622
Age at the time of diagnosis, years, Mean (SD)	33.9 (13.6)	38.8 (16.1)	< 0.001
Disease duration, years, Mean (SD)	18.4 (7.96)	4.1 (2.68)	< 0.001
Lichtiger index at diagnosis, Mean (SD)	3.05 (2.4)	3.25 (2.8)	0.393
*Treatment*			
Biologics and small molecule agents	53 (21.6%)	90 (32.1%)	0.007
IFX (n [%])	28 (11.4%)	37 (13.2%)	0.535
IFX-BS (n [%])	15 (6.1%)	23 (8.2%)	0.356
ADA (n [%])	18 (7.3%)	28 (10.0%)	0.283
GLM (n [%])	5 (2.0%)	20 (7.1%)	0.006
VED (n [%])	1 (0.4%)	5 (1.8%)	0.138
Tofacitinib (n [%])	5 (2.0%)	14 (5.0%)	0.069
*Other treatment*			
5-ASA (n [%])	226 (92.2%)	246 (87.9%)	0.096
Corticosteroids	165 (67.3%)	179 (63.9%)	0.411
Immunomodulator (n [%])	104 (42.4%)	96 (34.3%)	0.055
Calcineurin inhibitor (n [%])	44 (18.0%)	37 (13.2%)	0.141
Apheresis (n [%])	80 (32.7%)	65 (23.2%)	0.014
Probiotics (n [%])	191 (78.0%)	209 (74.6%)	0.519
Herbal drug (n [%])	30 (12.2%)	27 (9.6%)	0.342

In our cohort, anti-TNF-α antibodies were selected as the biologics of the first and subsequent lines in most cases of both UC and CD. Loss of response was more common as the reason of the discontinuation of the first line biologics in UC in the biologic era group (prebiologic era group vs. biologic era group; 8.0% vs. 25.3%, *P* = 0.020), while the discontinuation of the first line biologics due to adverse events was more frequent in UC in the prebiologic era (44.0% vs. 16.9%, *P* = 0.001). On the other hand, loss of response and adverse events in CD were similarly observed between the prebiologic era and biologic era groups as the reason of the discontinuation of the first line biologics. Concomitant use with immunomodulators was common in UC and CD in both eras (Suppl. Table 1).

### Association analysis for identifying unique therapeutic combinations in patients with UC

The association analysis was performed to show differences between the prebiologic and biologic era groups in the choice of drugs in patients who required multiple drugs. The analysis of the overall population of registered patients with UC in the Far East cohort revealed high lift values for VED selection in patients with multiple drug use histories (Supplementary Figure 3a). The association plots of the top 10 treatment combinations sorted by lift value (Supplementary Figure 3b) indicate drug combinations, and each multiple drug combination formed a cluster that was selected for VED.

**Figure 4:**
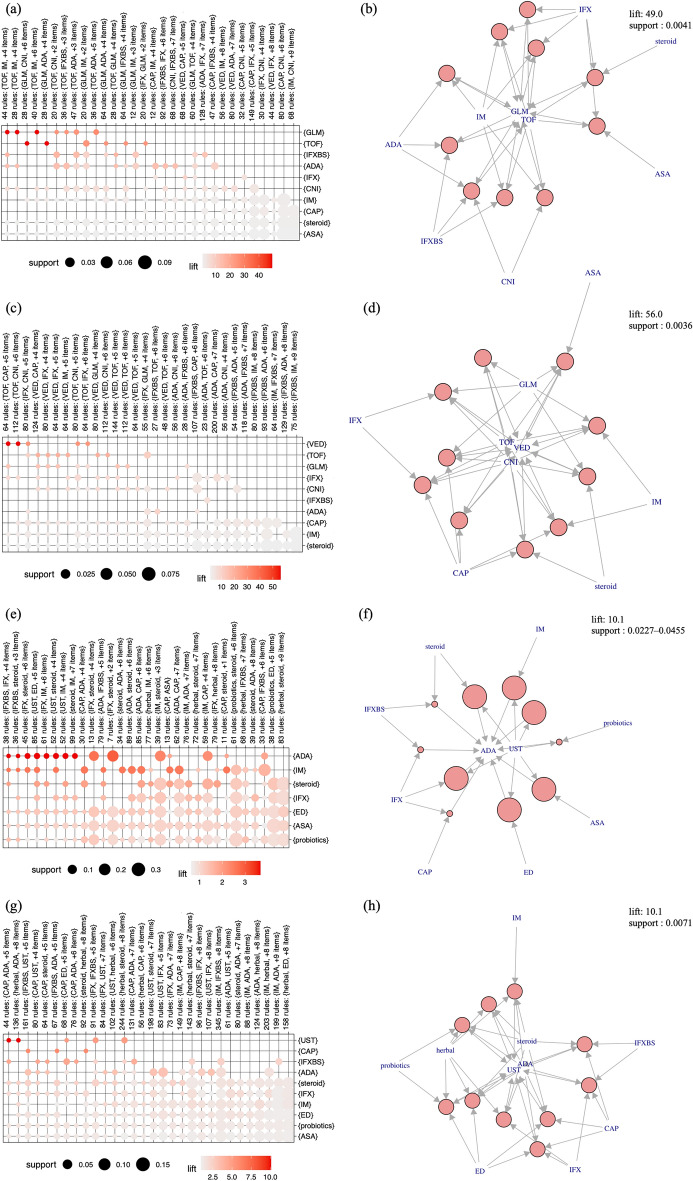
Association analysis in the patients with IBD who were diagnosed in the prebiologic and biologic eras. (**a**) The grouped matrix-based visualization combination in patients with UC diagnosed in the prebiologic era. In a balloon plot with antecedent medication groups as columns and consequent drugs as rows, the color represents the aggregated lift in the group and the size shows the aggregated support. The numbers of antecedent medication combinations with the names of the most frequently appeared drugs are displayed as the labels in the columns. The combinations between antecedent groups and a consequent drug with the highest lift value were placed in the upper left of this matrix, and the combinations were placed in the lower right according to the decrease in the lift value. (**b**) The rank of the top 10 treatment combination in patients with UC diagnosed in the prebiologic era (see description of the plot in Suppl. Figure 5). Each disc indicates the combination between an antecedent medication and a consequent drug with a top 10 high lift value. The support value was 0.0041 and the lift value was 49.0. (**c**) The grouped matrix-based visualization combination in patients with UC diagnosed in the biologic era. (**d**) The rank of the top 10 treatment combination in patients with UC diagnosed in the biologic era. The support value was 0.0036 and the lift value was 56.0. (**e**) The grouped matrix-based visualization combination in patients with CD diagnosed in the prebiologic era. (**f**) The rank of the top 10 treatment combination in patients with CD diagnosed in the prebiologic era. The disc size indicates the support value (0.0227–0.0455) and the lift value was 10.1. (**g**) The grouped matrix-based visualization combination in patients with CD diagnosed in the biologic era. (**h**) The rank of the top 10 treatment combination in patients with CD diagnosed in the biologic era. The support value was 0.0071 and the lift value was 10.1.

Next, patients who needed to use multiple drugs were likely to receive GLM with high lift values in the prebiologic era group (Fig. 4a, b), while VED was the most preferred agent in the biologic era group (Fig. 4c, d) when evaluating the differences in treatment combinations between the groups of patients diagnosed in both eras.

### Association analysis for identifying unique therapeutic combinations in patients with CD

The analysis of the overall population of registered patients with CD in the Far East cohort revealed high lift values for UST selection in patients with multiple drug use histories (Supplementary Figure 4a). The association of the top 10 treatment combinations sorted by lift value also revealed that UST was more likely to be prescribed in the situation of multiple treatment histories (Supplementary Figure 4b). ADA formed clusters with high lift values in the group of patients diagnosed in the prebiologic era (Fig. 4e, f), while UST was preferred with high lift values in the biologic era group (Fig. 4g, h) when evaluating the differences in treatment combinations between the groups of patients diagnosed in both eras.

## Discussion

The present study was conducted to clarify the trends of patients with UC and CD in Japan in the biologic versus the prebiologic era through the analysis of cases enrolled in the Far East 1000 cohort. Most hospital-based cohort studies of IBD include specialized hospitals for IBD alone, and those studies could not accurately reflect real-world practices because a considerable number of patients with IBD are treated at nonexpert hospitals, and physicians’ practices may differ between expert and nonexpert hospitals. Therefore, this cohort represents more credible real-world data due to the inclusion of nonexpert hospitals. Our cohort analysis showed not only the increased number of patients but also the difference in treatment strategy between patients with onset before and after biologic implementation.

The Far East 1000 includes patients from not only IBD expert hospitals but also from many general hospitals. Guidelines have been established for IBD treatment^9^, and nonexperts are now able to select treatment following these guidelines. However, our results revealed that nonexperts mainly manage relatively easy UC cases that could be treated with 5-ASA and/or corticosteroids, while steroid-resistant or steroid-dependent cases with immunomodulator and/or biologic administration are mostly managed at expert hospitals. Hence, most CD cases were managed by experts because approximately 80% of patients with CD received biologics. The difficulty in the medical management of CD, including handling small intestinal lesions that can easily develop perforation and abscess formation, may also affect the dominance of treatment by specialists^20^. Our findings suggest that most nonexperts could not appropriately apply novel drugs with various mechanisms of action to patients with refractory IBD and there must be a gap at the medical level between hospitals with and without IBD specialists. Providing optimal medical care would be even more difficult for nonspecialists because more new drugs are expected to be launched. Therefore, appropriate education for nonspecialists, interhospital collaboration, and training and placement of IBD specialists are eagerly required to fill the gap between hospitals with and without specialists.

The implementation of biologics in IBD treatment has brought about a revolution in outcomes and strategies^21,22^. Our study revealed that both patients with UC with onset at both the prebiologic and biologic era were treated with a variety of biologic agents. However, the use of conventional therapies, such as immunomodulators and apheresis, was relatively more frequent in the prebiologic era group, while the use of biologics was significantly more frequently observed in the biologic era group. The biologic era group was likely to show more diversity in the selection of the biologic agent options, while anti-TNF-α agents were more likely to be indicated for patients with multiple treatment histories in the prebiologic era group (Fig. 4c–f) for both UC and CD. Thus, the progress of medical treatment in IBD has changed the trend of treatment choice. These results might reflect the true trend of medical preference in both patients and physicians because the Japanese unique subsidy system enables to use of expensive but highly effective drugs, such as biologics, without considering cost-effectiveness^23^.

The association analysis of patients with UC who received two or more medications revealed that GLM was the preferred drug in the prebiologic era group, while VED was likely to be selected in the biologic era group. The preference for VED in the biologic era may be associated with the recent increase in patients with UC with older onset^24^, as shown in our results. Theoretically, VED does not suppress patient immunity and is considered safe and suitable for older patients, and physicians may prefer VED to other biologics for older patients^25^. Therefore, the recent trend of UST prescription after its approval for UC is interesting because clinical practice guidelines state that UST is considered a safe drug for older patients^26^.

The prebiologic era group relatively favored ADA, while the frequency of UST selection was relatively increased in the biologic era group for the choice of biologic agents in patients with CD. IFX has been used first for moderate to severe CD and ADA was the second approved biologics since the IFX approval in 2002 in Japan. Hence, ADA had been preferred not only in cases refractory to conventional therapies, such as corticosteroids and immunomodulators but also in cases refractory to IFX, suggesting that patients in the prebiologic era who had to be treated with various therapeutic options were more likely to receive ADA. A meta-analysis conducted before the UST approval revealed that ADA was effective for induction and remission maintenance^27^.

Patients with CD who received UST were more likely to have been administered anti-TNF-α, such as IFX or ADA, in their treatment history (Fig. 4h), reflecting the increased options of biologic agents. A variety of treatment options are available for patients with CD who are diagnosed in the biologic era, but the results of head-to-head trials between different biologics have remained insufficient to determine the optimal therapeutic strategy. A recent study with a comparison of the efficacy between UST and ADA in CD showed no difference in clinical remission at 52 weeks between the two biologics^28^. A network meta-analysis of patients with moderate to severe CD previously treated with anti-TNF-α, which was conducted after the introduction of antiIL23 agents, showed a preference for a switch to antiIL23 or another anti-TNF-α in nonresponders to prior anti-TNF-α^29^. Our real-world data indicated that UST may be preferred in situations after using both two anti-TNF-α agents and appears to be consistent with the clinical trial and meta-analysis results.

This is a hospital-based registry study with the not so large sample size. Therefore, there might be a limitation of having a bias in patient selection compared to population studies. In addition, due to the short observation period, life expectancy could not be analyzed in this study. Also, this study was performed based on the data registered in 2019, and the observation period for UC in the biological era was particularly short. However, this cohort appears to provide real-world data on patients undergoing IBD treatment because it covers both IBD experts and community hospitals in the region with low patient mobility.

In conclusion, this study demonstrated the characteristics of patients with IBD in a special region of Japan with minimal patient mobility. The onset age has recently got older in both UC and CD cases. Additionally, therapeutic strategies have changed with various biologic applications. These results indicate that the increasing diversity of both patient characteristics and therapeutic agents requires the establishment of more individualized treatment strategies.

### Supplementary Information

Supplementary Information 1.

Supplementary Information 2.

Supplementary Information 3.

Supplementary Information 4.

Supplementary Information 5.

Supplementary Information 6.

## Data Availability

The datasets used and analysed in the current study available from the corresponding author on reasonable request. IRB did not permit data sharing, because we did not inform patients of data sharing.

## Abbreviations

IBD: Inflammatory bowel disease
UC: Ulcerative colitis
CD: Crohn’s disease
TNF: Tumor necrosis factor
IFX: Infliximab
IFX-BS: Infliximab-biosimilar
ADA: Adalimumab
GLM: Golimumab
UST: Ustekinumab
VED: Vedolizumab
TOF: Tofacitinib
5-ASA: 5-Aminosalicylic acid
IM: Immunomodulator
CAP: Cytapheresis
ED: Elemental diet
CNI: Calcineurin inhibitor
