# Supplementary material for: Treatment strategy changes for inflammatory bowel diseases in biologic era: results from a multicenter cohort in Japan, Far East 1000
Source: Sci Rep. 2023 Aug 21;13:13555. doi: 10.1038/s41598-023-40624-5 (PMC10442357; doi:10.1038/s41598-023-40624-5)
Supplement: Supplementary file 1 — Supplementary Information 1. [file 41598_2023_40624_MOESM1_ESM.pdf]

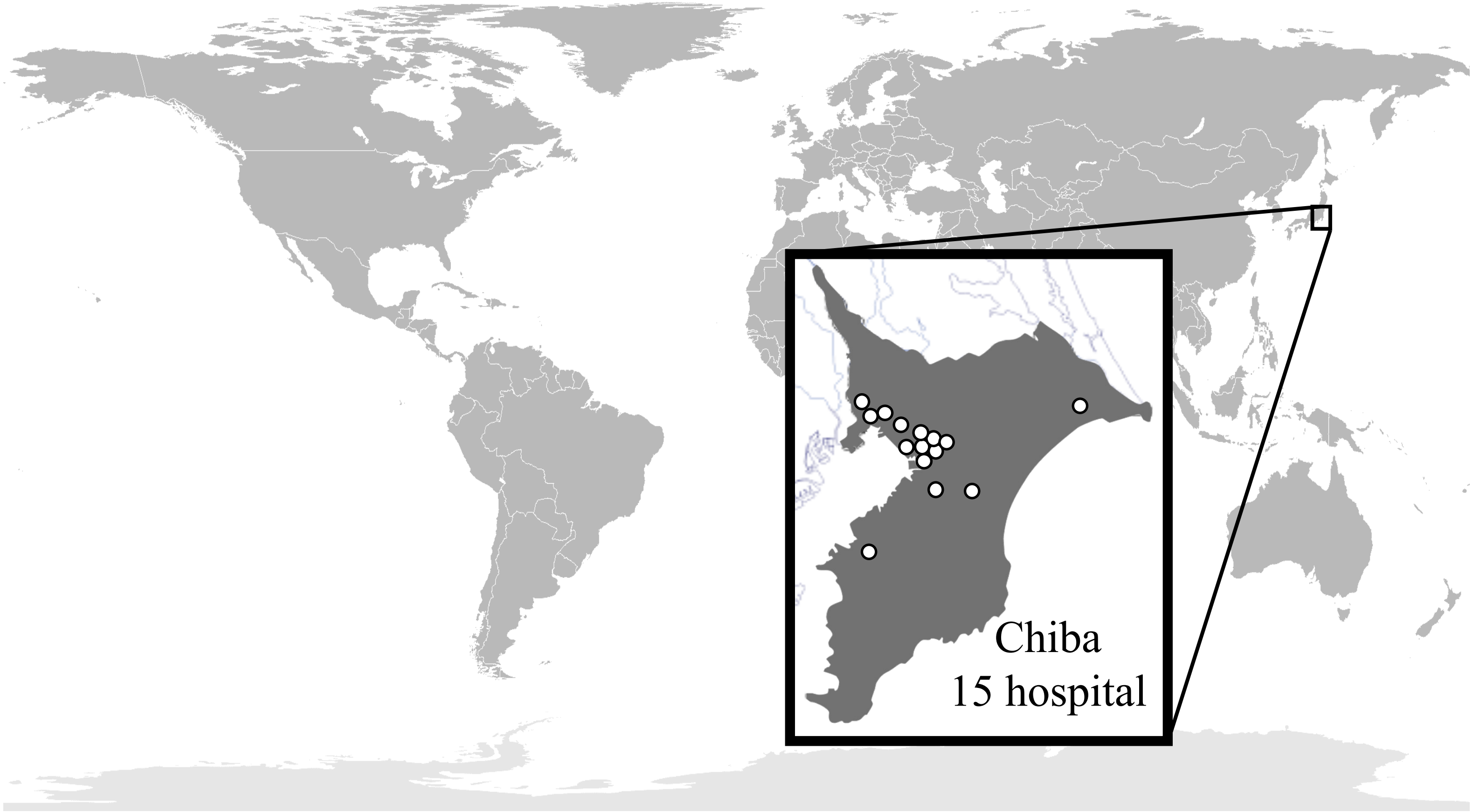


**Supplementary Figure 1. The geographical areas and location of hospitals in the Far East 1000 cohort study.**

The hospital-based cohort in the Chiba prefecture, Japan in 2018–2020. The geografphical figure was created using Microsoft PowerPoint software (version 16.74) with the obtained maps (https://en.wikipedia.org/wiki/File:BlankMap-World6-Equirectangular.svg, https://d-maps.com/carte.php?num_car=11163&lang=en).
