# Supplementary material for: Treatment strategy changes for inflammatory bowel diseases in biologic era: results from a multicenter cohort in Japan, Far East 1000
Source: Sci Rep. 2023 Aug 21;13:13555. doi: 10.1038/s41598-023-40624-5 (PMC10442357; doi:10.1038/s41598-023-40624-5)
Supplement: Supplementary file 2 — Supplementary Information 2. [file 41598_2023_40624_MOESM2_ESM.pdf]

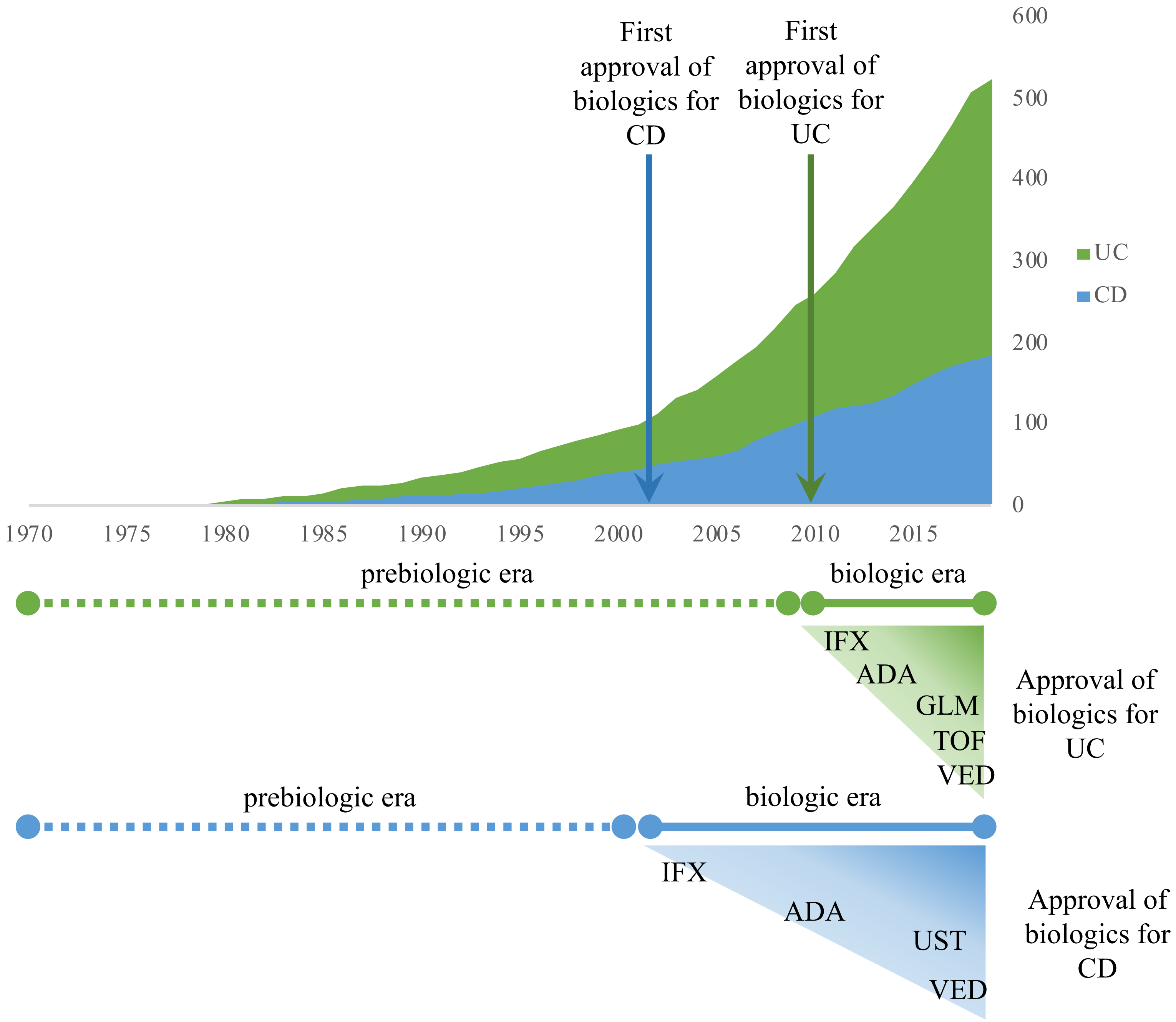

Supplementary Figure 2. Numbers of patients with disease diagnosis according to calendar year. In Japan, the first approval of biologics was in 2002 for CD and in 2010 for UC.

This study defined the prebiologic era as the period until the first approval of biologics in Japan for either disease and the biologic era as the period after the approval of biologics.
