# Supplementary material for: Treatment strategy changes for inflammatory bowel diseases in biologic era: results from a multicenter cohort in Japan, Far East 1000
Source: Sci Rep. 2023 Aug 21;13:13555. doi: 10.1038/s41598-023-40624-5 (PMC10442357; doi:10.1038/s41598-023-40624-5)
Supplement: Supplementary file 3 — Supplementary Information 3. [file 41598_2023_40624_MOESM3_ESM.pdf]

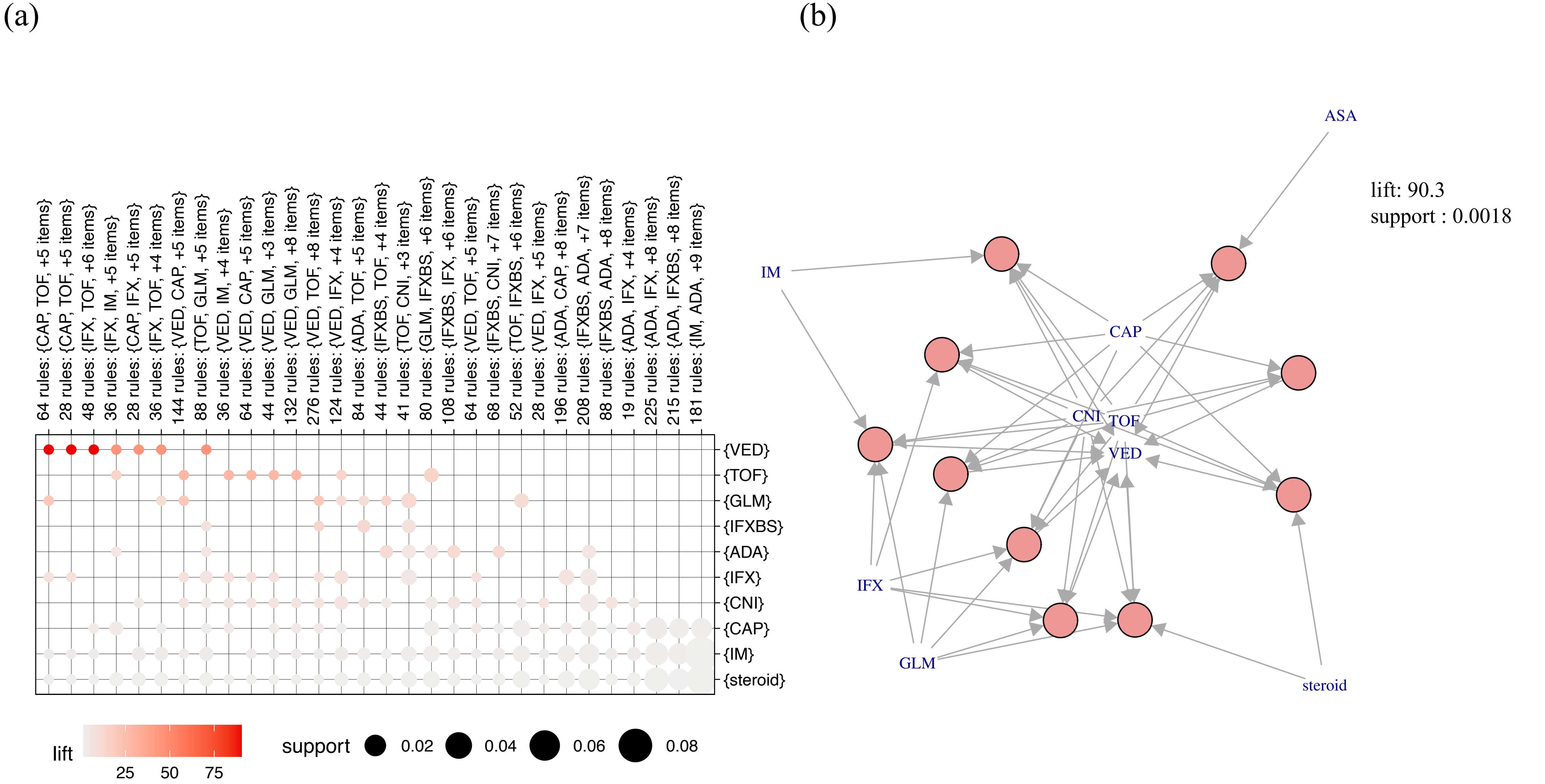

Supplementary Figure 3. The association analysis in all patients with UC who were recruited in the Far East 1000 cohort.
(a) The grouped matrix-based visualization combination. In balloon plots with antecedent medication groups as columns and consequent drugs as rows, the color represents the aggregated lift in the group and the size shows the aggregated support. The numbers of antecedent medication combinations with the names of the most frequently appearing drugs are displayed as labels in the columns. The combinations between antecedent groups and a consequent drug with the highest lift value were placed in the upper left of this matrix, and the combinations were placed in the lower right according to the decrease in the lift value.
(b) The rank of the top 10 treatment associations was shown in the plot (see description of the plot in Suppl. Fig.1). Each disc indicates the combination between an antecedent medication and a consequent drug with a top 10 high lift value. The support value was 0.0018 and the lift value was 90.3.
