# Supplementary material for: Treatment strategy changes for inflammatory bowel diseases in biologic era: results from a multicenter cohort in Japan, Far East 1000
Source: Sci Rep. 2023 Aug 21;13:13555. doi: 10.1038/s41598-023-40624-5 (PMC10442357; doi:10.1038/s41598-023-40624-5)
Supplement: Supplementary file 5 — Supplementary Information 5. [file 41598_2023_40624_MOESM5_ESM.pdf]

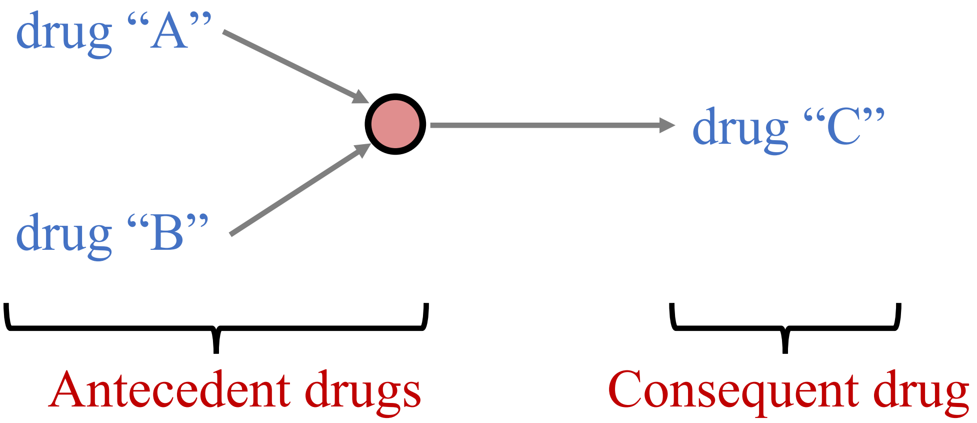

Supplementary Figure 5. The description of antecedent and consequent drugs in the plot regarding grouped matrix-based visualization combination.
In this plot, antecedent drug sets are shown as red discs where each drug is clustered with an arrow, and the consequent drug is located at the arrowhead from the disc.
