# Supplementary material for: Treatment strategy changes for inflammatory bowel diseases in biologic era: results from a multicenter cohort in Japan, Far East 1000
Source: Sci Rep. 2023 Aug 21;13:13555. doi: 10.1038/s41598-023-40624-5 (PMC10442357; doi:10.1038/s41598-023-40624-5)
Supplement: Supplementary file 6 — Supplementary Information 6. [file 41598_2023_40624_MOESM6_ESM.docx]

**Supplementary Table 1. The response to biologics of the first and subsequent lines in patients with ulcerative colitis and Crohn’s disease**

|  |  | **UC** |  |  |  | **CD** |  |
| --- | --- | --- | --- | --- | --- | --- | --- |
|  | **prebiologic era** | **biologic era** | **P value** |  | **prebiologic era** | **biologic era** | **P value** |
| **1st line biologics** | **50** | **83** |  |  | **36** | **110** |  |
| **Anti-TNF-α (%)** | **49 (98.0%)** | **77 (92.8%)** | **0.254** |  | **36 (100%)** | **106 (96.4%)** | **0.572** |
| **Other biologics (%)** | **1 (2.0%)** | **6 (7.2%)** | **0.254** |  | **0 (0.0%)** | **4 (3.6%)** | **0.572** |
| **Concomitant therapy with immunomodulator (%)** | **41 (82.0%)** | **57 (68.7%)** | **0.107** |  | **16 (44.4%)** | **62 (56.4%)** | **0.250** |
| **Primary non-response of 1st line biologics (%)** | **3 (6.0%)** | **7 (8.4%)** | **0.742** |  | **0 (0.0%)** | **3 (2.7%)** | **1.000** |
| **Loss of response of 1st line biologics (%)** | **4 (8.0%)** | **21 (25.3%)** | **0.020** |  | **11 (30.6%)** | **38 (34.5%)** | **0.691** |
| **Adverse events with 1st line biologics (%)** | **22 (44.0%)** | **14 (16.9%)** | **0.001** |  | **4 (11.1%)** | **19 (17.3%)** | **0.442** |
| **Switch to 2nd line biologics** | **16** | **28** |  |  | **9** | **42** |  |
| **Anti-TNF-α (%)** | **16 (100%)** | **28 (100%)** | **0.852** |  | **9 (100%)** | **32 (76.2%)** | **0.676** |
| **Other biologics (%)** | **-** | **-** |  |  | **0 (0%)** | **10 (23.8%)** | **0.120** |
